# Parental technoference and adolescents’ mental health and violent behaviour: a scoping review

**DOI:** 10.1186/s12889-023-16850-x

**Published:** 2023-10-19

**Authors:** Donna Dixon, Catherine A. Sharp, Karen Hughes, J. Carl. Hughes

**Affiliations:** 1 School of Educational Sciences, CIEREI, Bangor University, Bangor, Gwynedd, UK; 2 Public Health Collaborating Unit, School of Medical and Health Sciences, College of Human Sciences, Bangor University, Wrexham Technology Park, Wrexham, UK; 3 Policy and International Health, World Health Organization Collaborating Centre on Investment for Health and Well-Being, Public Health Wales, Wrexham, UK

**Keywords:** Technoference, Phubbing, Parent, Adolescent, Mental Health, Violent Behaviour

## Abstract

**Purpose:**

The term ‘technoference’ refers to habitual interferences and disruptions within interpersonal relationships or time spent together due to use of electronic devices. Emerging evidence suggests associations between parental technoference and young people’s mental health and violent behaviours. This scoping review sought to summarise the existing literature.

**Methods:**

A scoping review was undertaken across six databases (APA PsycINFO, MEDLINE, ASSIA, ERIC, Social Sciences Premium Collection, SciTech Premium). Searches included articles examining the association between parental technoference and adolescent mental health and violent behaviours. All included studies provided empirical findings.

**Results:**

Searches retrieved 382 articles, of which 13 articles met the eligibility criteria. A narrative approach was applied to synthesise the eligible findings. Across all studies, adolescent perceptions of parental technoference were negatively associated to adolescent mental health and positively related to adolescent violent behaviours. Parental cohesion and mental health were identified as significant mediating factors.

**Conclusion:**

Findings suggest that parents should be aware of the environment in which they use electronic devices as their use can potentially, directly and indirectly, influence adolescent mental health and violent behaviours. Further research into the potential caveats of parental technoference could support the development of evidence-informed guidelines for parental management of electronic devices.

**Supplementary Information:**

The online version contains supplementary material available at 10.1186/s12889-023-16850-x.

Digitalisation within contemporary society has enabled electronic devices such as smartphones, tablets, laptops and games consoles to permeate family life. Technical advances in internet connectivity alongside device portability has increased ownership of mobiles and allowed continuous engagement and connectivity [1]. In particular, there has been a surge in the uptake of smartphones over the last decade, reaching over half of the world’s population [2]. For instance, in the USA, smartphone use in those aged 18 + rose from 35–85% between 2011 and 2021 [3], whilst in China 1.22 billion people had subscribed to mobile services by 2021, representing 83% of the population [4]. Despite the benefits technology has created for adults, such as increased social support [5] and the flexibility to work from home [6], research highlights the potential for disruption of in-person social dynamics by mobile and digital technology use. Initially, this demeanour was dubbed ‘absent presence’; referring to an individual being physically present but being distracted by communication or mobile content [7]. The term ‘technoference’ was adopted to describe habitual interruptions in interpersonal relationships or time spent together caused by technological devices [8, 9]. Similarly, the term ‘phubbing’, blending the words ‘phone’ and ‘snubbing’ is used to characterise a direct disregard for another individual in favour of one’s phone [10]. Both terms illustrate that uninhibited device use during interactions with others can result in social exclusion and interpersonal neglect.

Studies on technoference were initiated in romantic relationships finding that diminished interactions due to digital interruptions led to greater conflict between couples and lower relationship satisfaction, resulting in depression and lower life satisfaction [9, 11]. However, research has since begun to explore the association between technoference and the parent-child dynamic, reporting the extent of electronic device use within families and its potential impairment on parent-child interactions [12], parenting quality [13] and children’s behaviour [14]. The emergence of digital distraction could be worse than non-digital distractions due to the strong habits or addictive behavioural tendencies devices can elicit [12]. Early research suggests that breaking attention with digital devices is more challenging than other parental distractions, such as reading, eating or chatting [15] as there is no prescribed end point to the activity. Consequently, a child’s needs and cues for attention are less likely to be met [15–17]. However, evidence on the strength of the association between people and their addiction to digital devices is currently being debated in the literature [18].

Given the multifaceted features of digital devices, parents report how emotionally connected they are to their device, experiencing difficulty in disconnecting digitally [19]. Parents also express their anxiety of being without their mobile phone, reporting the fear of missing out and the pressure to respond to work commitments [1]. Parents reported using electronic devices during family time, such as at home [16], during meal times [20] and at playgrounds [13, 21]. They also reported being less attentive and responsive to their young children when immersed in electronic devices, with fewer verbal and non-verbal parent-child interactions [15, 16, 22]. Consequently, it is argued that parental technoference in public is a safety risk to children due to decreased parental awareness and supervision, which in turn can increase child injuries [22, 23]. Further, observations suggest parents can demonstrate less sensitivity towards their children when digitally distracted, using harsher and angry parenting styles [12, 24, 25]. Parents also describe feeling distracted due to frequent device use, and this resulting in diminished connection and cohesion with their children [13, 15, 20, 24]. Owing to their own technoference, parents have reported negative behaviours in children, such as whining and sulking [14], being less relaxed, and more emotional and unsatisfied [23, 24]. Similarly, surveys also reported positive associations between parental technoference and violent behaviours in young children (< 10 years), such as physical aggression [26].

The majority of existing research examining parental technoference has focused on younger children (< 12 years), and been conducted in the USA. Previous reviews have summarised the evidence on the relationship between parental technoference and younger children’s behavioural outcomes and on parent-child interactions [16, 22, 27–29]. However, at the point of undertaking, no reviews had explored outcomes for adolescents in this context. Due to the cognitive and psychological development that occurs during adolescent years [30], it is important to understand the impact of technoference during these years. Two fundamental areas of adolescence are mental health and exhibiting violent behaviours. For example, the World Health Organization recognises the adolescent years as the lifetime period that mental health difficulties are most prevalent [31]. Poor mental health in adolescence can include depression [32], anxiety [33] and addiction [34], and evidence has found mental health condition tracks into adulthood [31]. In addition, youth violence which includes a range of acts from bullying to physical fighting, is a global public health concern and can pose long terms impacts on health and well-being [35]. Adolescents report their frustration at parental device use interrupting valuable family time, their expectations of parents to refrain from using digital devices during family time, and that they perceive parents as being less responsive whilst using their devices [17, 36, 37].

Addressing the knowledge gap, to the best of our knowledge, this review is the first to synthesise research on the association between parental technoference and adolescent mental health and violent behaviour. For the purpose of this review, mental health includes both mental health difficulties (e.g. anxiety, depression, addiction), and well-being (e.g. self- esteem, social sensitivity, life satisfaction). In contrast to a systematic review approach which aims to explore the effectiveness of a treatment or practice, a scoping review methodology was applied to summarise existing research and knowledge in the area and to identify gaps to inform future research [38].

## Methods

### Research Question

Research questions for this review are:

(1) What is known about the association between parental technoference and adolescent mental health outcomes?

(2) What is known about the association between parental technoference and adolescent violent behaviours?

### Procedure

#### Identifying relevant studies

This review followed the Preferred Reporting Items for Systematic Reviews guidelines (PRISMA; see Supplementary Table 1) [39]. Using the ProQuest platform, a systematic search for peer-reviewed studies was undertaken across six databases (APA PsycINFO, MEDLINE, ASSIA, ERIC, Social Sciences Premium Collection, SciTech Premium). Search terms are listed in Table 1. The search was conducted in the English language. No restrictions were placed on publication dates (due to recency of the research area), or applied on the geographical location, setting of enquiry, method for enquiry (e.g., self-report) or data collection tool (e.g. questionnaires, interviews). The search was conducted by the lead author (DD) in October 2021 and retrieved 382 unique references.

**Table 1:** Search terms entered into the ProQuest database

Technology Terms	Parent Terms	Outcome
TIAB(technoference OR	TIAB(parent* OR	TIAB(violence OR
phubbing OR	maternal OR	bullying OR
distracted OR	paternal OR	cyberbullying OR
smart*phone OR	mother OR	aggress* OR
“mobile phone” OR	father OR	addiction OR
“mobile device”)	caregiver))	depress* OR
		anxiety OR
		“mental health“ OR
		“mental* ill*” OR
		devian* OR
		problem OR
		behav*)))

#### Study selection

Results were inputted to Microsoft Excel. Title and abstracts from all reviewed references were assessed independently by the first author (DD) and a second reviewer (KH: 40%; CAS: 30%; NW: 30%) to ascertain eligibility for inclusion. Disagreements between reviewers were resolved by a third reviewer. The eligibility of studies was confirmed according to their adherence to the following inclusion criteria: (a) published in peer-reviewed journals; (b) present primary data on the association between parental technoference or parental phubbing and adolescents (i) mental health (e.g., depression, anxiety, addiction) in adolescents, and/or (ii) violent behaviours (e.g., aggression, bullying, risk-taking); and (c) present data for populations between the ages of 10–19 years, in accordance with the World Health Organisations definition of adolescents (samples were included if the majority of the participants were within this age range) [31].

#### Charting the data

**Fig. 1:**
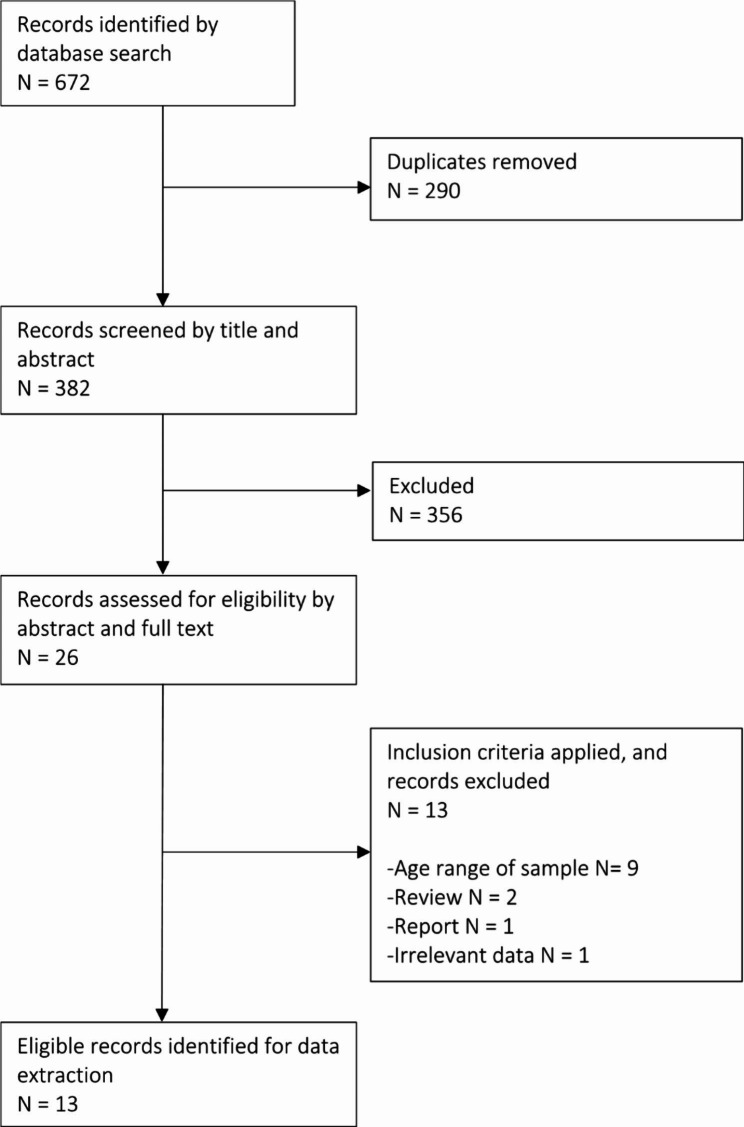
PRISMA flow diagram of study selection

A total of 26 articles were selected for full-text review by two independent reviewers (DD and KH), of which 13 were identified for inclusion (see Fig. 1). Extracted data included specific information on the authors; year of publication; country; study type; setting; sample size; age range or mean age of participants; measurement tools; study aim; mediating factors; theories which underpinned each study; and key findings relevant to the research questions. Data was narratively synthesised, which involved generating thematic descriptive accounts and evidence tables, presented separately for each outcome category, outlined in Tables 2 and 3. This approach allowed for an overview of the literature on the association between parental technoference and adolescent mental health and violent behaviours.

**Table 2:** Characteristics of included studies

							Outcomes explored			Mediators	
Author	Country	Study type	Setting	Sample size	Sample age (years)	Parental technoference measurement tool	Adolescent techno-ference	Mental health	Violent behaviour	Relationship quality	Psychological Factors
Bai et al., 2020	China	Cross-sectional	School	2,996	Mean age 16	Generic Scale of Being Phubbed	√	√			Agreeableness / Neuroticism
Bai et al., 2021	China	Cross-sectional	School	3,322	Mean age 16	Generic Scale of Being Phubbed		√		√	Depression
Geng et al., 2021	China	Cross-sectional	School	1,447	Mean age 16	Generic Scale of Being Phubbed		√			Loneliness / Fear of Missing Out
Liu et al., 2020a	China	Cross-sectional	School	303	12–16	The Technoference Scale		√		√	Life Satisfaction
Liu et al., 2020b	China	Cross-sectional	School	3,051	Mean age 13	Parental Phubbing Scale		√			Social sensitivity / Loneliness
Qu et al., 2020	China	Cross-sectional	School	4,213	10–20	Generic Scale of Being Phubbed			√	√	Perceived Mother Acceptance
Stockdale et al., (2018)	USA	Cross-sectional	School	1,072	10–20	The Technoference Scale	√	√	√	√	Anxiety / Depression
Wang et al., 2020	China	Longitudinal	School	2,407	Mean age 12	Parental Phubbing Scale			√	√	Low Self Esteem / Perceived Social Support
Wang et al., 2020	China	Longitudinal	School	2,407	Mean age 12	Parental Phubbing Scale		√			Moral Disengagement
Wei et al., 2021	China	Cross-sectional	School	874	11–18	Parental Phubbing Scale		√	√	√	Anxiety
Xie et al., 2019	China	Cross-sectional	School	1,007	11–16	Parental Phubbing Scale		√	√	√	Mobile Phone Addiction
Xie & Xie, 2020	China	**Study 1** Cross-sectional **Study 2** Cross-sectional	School School	530 293	**Study 1** Mean age 13 **Study 2** Mean age 12	Parental Phubbing Scale		√			Relatedness / Life Satisfaction
Zhang et al., 2021	China	Cross-sectional	School	471	Mean age 13	Parental Phubbing Scale		√		√	Social Anxiety / Core Self Evaluation

Generic Scale of Being Phubbed (Chotpitayasunondh & Douglas, 2018); The Technoference Scale (McDaniel & Coyne, 2016); Parental Phubbing Scale (Roberts & Davies, 2016)

**Table 3:** Summary of methods and findings exploring the association between parental technoference and adolescent mental health

Citation	Study aim	Theory	Key findings
Bai et al., 2020	To understand the association between mother phubbing, adolescent academic burnout and the moderating role of mental health.	Displacement Hypothesis; Diathesis-Stress Model	Mother phubbing was positively associated with children’s academic burnout through poor mental health. The relationship between mother phubbing and adolescent mental health was moderated by agreeableness, and neuroticism aggravated the influence of general mental health on academic burnout.
Bai et al., 2021	To explore whether parental phubbing would be positively related to adolescent phubbing and whether this would be positively related to adolescent depressive symptoms and the mediating role of attachment avoidance.	Displacement Hypothesis; Person–Environment Hypothesis	Parental phubbing was positively associated with adolescent phubbing as well as depressive symptoms. Attachment avoidance moderated the congruence and incongruent effects on parent/adolescent phubbing on adolescent depressive symptoms.
Geng et al., 2021	To examine the relationship between early perceived parental phubbing and subsequent problematic smartphone use and the mediating factors of loneliness and fear of missing out.	Social Learning Theory; Compensatory Internet Use Theory	Parental phubbing predicted adolescents’ subsequent problematic smartphone use. Loneliness and fear of missing out sequentially mediated the relationship.
Liu et al., 2020a	To examine the effect of parental phubbing on adolescent life satisfaction and addressing the role of the parent adolescent relationship and adolescent attachment styles.	Social Rejection Theory; Assets Theory	The conditional effect of parental phubbing on adolescents’ life satisfaction was significant among the preoccupied teens and the fearful teens but not significant among the secure teens and the dismissing teens.
Liu et al., 2020b	To explore the association between parental technoference and adolescent smartphone addiction and the mediating effects of social sensitivity and loneliness.	Ecological Systems Theory; Risky Families Model	Parental technoference could positively predict adolescent social sensitivity and loneliness and in turn social sensitivity and loneliness were positively associated with smartphone addiction tendency.
Stockdale et al., 2018	To examine the direct relationship among adolescents’ perceptions of parent-adolescent technoference and the impact on adolescent depression, anxiety, cyberbullying pro-social behaviour and civic engagement.	Attachment Theory	Parental technoference was associated with adolescent technoference which were uniquely related to increased anxiety, depression as mediated through parental warmth.
Wang et al., 2020(a)	To examine whether self-esteem and perceived social support would simultaneously moderate the relationship between parental phubbing and adolescent depressive symptoms.	Family Systems Theory	Adolescents with a high level of parental phubbing were likely to have a high level of depressive symptoms. Higher levels of parental phubbing significantly predicted depressive symptoms when adolescent self-esteem and perceived social support were low.
Xie et al., 2019	To determine if adolescent mobile phone addiction increases after being phubbed by parents and examine effects of the mediating roles of parent child attachment, deviant peer affiliation and moderating role of gender.	Social Control Theory; Informal Social Control Theory	Parental phubbing was positively related with adolescent mobile phone addiction. Parent-child attachment and deviant peer affiliation was found to mediate the relationship.
Xie & Xie, 2020	To test the connections between parental phubbing and depression in late childhood and adolescence as well as the mediating roles of parental warmth parental rejection and relatedness need satisfaction.	Expectancy Violations Theory; Self-Determination Theory	Parental phubbing was associated with adolescents’ depressions in both studies. Mediating factors included parental warmth, relatedness and satisfaction.
Zhang et al., 2021	To examine the potential mechanism between parental phubbing and adolescent mobile phone addiction and the mediating role of social anxiety and core self-evaluations.	Social Learning Theory	Social anxiety and core self-evaluation played multiple roles in the association between parental phubbing and adolescent mobile phone addiction, with parental phubbing influencing adolescent mobile phone addiction.

## Results

### Study characteristics

Table 2 provides an overview of the characteristics of each eligible study. All studies were quantitative and collected data from young people using self-report questionnaires in secondary school settings. Three self-reported parental technoference outcome measures were identified and reliability reported. These were modified versions of The Technoference Scale (*a* = 0.87) [40], the Parental Phubbing Scale (*a* = 0.87) [41], and the Generic Scale of Being Phubbed Scale (*a* = 0.95) [42]. Articles were published over a three-year period (2018 and 2021), with the majority conducted in China (n = 12) and one in the USA. Studies measured adolescent perspectives on either parental phubbing (n = 11) or parental technoference (n = 2). Ten studies examined the association between parental technoference and adolescent mental health and five explored the relationship between parental technoference and adolescent violent behaviour. Table 2 shows the outcomes measured by each study. Only two studies reported the prevalence of technoference or phubbing among the adolescent samples; Stockdale et al. (2018) [40] found in 2016 that 77.5% of American adolescents reported parental technoference at least some of the time, whilst Liu et al. (2020a) [41] identified in 2019 that 87.5% of adolescents in China revealed that they experienced parental phubbing on a daily basis.

### Evidence on the association between parental electronic device distraction and adolescent mental health

Ten studies investigated relationships between parental technoference and adolescent mental health (see Table 3). Sample sizes ranged from 293 to 3,322 with an age range of 10–20 years. All studies reported a negative correlation between parental technoference and adolescent mental health.

The association between parental technoference and levels of adolescent depression and/or anxiety was explored in four studies [40, 42–44]. Greater perceived parental technoference was related to increased adolescent depression and anxiety in all studies. Two studies [40, 42] also investigated the association between parental technoference and adolescents’ own technoference patterns and subsequently how these affected levels of adolescent depression and anxiety. These studies found a positive correlation between parental technoference and adolescent technoference, which exacerbated levels of depression and anxiety, both independently and simultaneously. Furthermore, adolescent depression levels increased as adolescent and parental technoference increased [42]. One study described that adolescents who reported frequent parental technoference experienced lower levels of life satisfaction [47]. Additionally, a negative correlation between perceptions of technoference and adolescents’ mental health was found which subsequently predicted academic burnout [45]. The relationship between perceived parental technoference and levels of adolescent mobile phone addiction was also investigated in a further four studies [41, 46, 48, 49]. Across all studies, a positive correlation was identified between perceived parental technoference and adolescent addictive mobile phone use.

Six studies examining the association between parental technoference and adolescent mental health identified potential mechanisms underlying the associations (see Table 3). Firstly, characteristics of parent-adolescent relationships were repeatedly identified as predominant mediating factors. One study found that the association between parental technoference and adolescent mobile phone addiction was moderated by the quality of parent-adolescent attachment [48]. It was also revealed that the association between parental technoference and decreased adolescent life satisfaction was greater in adolescents who demonstrated preoccupied or fearful attachment styles [47]. Additionally, adolescent attachment avoidance was found to moderate the congruent and incongruent effects of parent adolescent technoference on adolescent depressive symptoms [42]. Parental warmth was also identified as a risk factor [40, 44], with lower levels of perceived parental warmth significantly predicting adolescent depression and/or anxiety. Further, it was reported that lower levels of perceived family social support was a mediating factor in the link between parental technoference and adolescent depressive symptoms [43].

Five studies also highlighted that the association between parental technoference and adolescent mental health can be dependent upon the psychological factors of adolescents (see Table 3). One study found a decline in mental health as a consequence of perceived parental technoference was moderated by adolescent agreeableness and neuroticism, with highly agreeable adolescents increasingly affected [45]. Similarly, low adolescent self-esteem was identified as a mediating factor between parental technoference and adolescent depressive symptoms [43]. Studies also found that the association between parental technoference and adolescent addictive mobile phone use was higher among adolescents who reported increased levels of loneliness, social sensitivity [41], fear of missing out [46], social anxiety and core self-evaluations [49].

### Evidence on the association between parental electronic device distraction on adolescent violent behaviour

Five studies explored associations between perceived parental technoference and adolescent violent behaviour (see Table 4). Sample sizes ranged from 424 to 4,213 with an age range of 10–20 years. Four studies examined relationships between perceived parental technoference and cyberbullying perpetration [40, 50–52], and one study investigated the role of parental technoference in adolescent deviant peer affiliation [48]. Findings highlighted that adolescents who frequently experienced parental technoference were more likely to engage in cyberbullying [50–52]. Similarly, it was reported that parent and adolescent technoference independently and simultaneously were predictive of adolescent cyberbullying [40]. The only study which examined parental technoference as a risk factor for deviant peer affiliation found a negative association, which subsequently mediated the development of adolescent mobile phone addiction [48].

**Table 4:** Summary of methods and findings for studies exploring the association between parental technoference and adolescent violent behaviours

Citation	Study aim	Theory	Key findings
Qu et al., 2020	To examine whether mother phubbing would be positively related to adolescent cyberbullying and if perceived mother acceptance or emotional stability mediates this relationship.	Displacement Hypothesis; Parental Rejection Theory	Mother phubbing was positively related to adolescent cyberbullying, which was mediated by perceived mother acceptance.
Stockdale et al., 2018	To examine the direct relationship among adolescents’ perceptions of parent-adolescent technoference and the impact on adolescent depression, anxiety, cyberbullying pro social behaviour and civic engagement.	Attachment Theory	Parental technoference was associated with adolescent technoference which were uniquely related to increased cyberbullying, mediated through parental warmth.
Wang et al., 2020(b)	To examine whether parental phubbing was significantly related to adolescent cyberbullying perpetration and if moral disengagement mediated this relationship.	Frustration Aggression Theory	Adolescents with a high level of parental phubbing were likely to cyberbully others. Moral disengagement significantly mediated the relationship between parental phubbing and adolescent cyberbullying perpetration.
Wei et al., 2021	To investigate the association between parental phubbing and adolescent cyberbullying perpetration and the mediating role of anxiety and Zhong-Yong thinking.	Social Control Theory	Parental phubbing was positively associated with adolescent cyberbullying perpetration and anxiety mediated this association.
Xie et al., 2019	To determine if adolescent mobile phone addiction increases after being phubbed by parents and examine effects of the mediating roles of parent child attachment, deviant peer affiliation and moderating role of gender.	Social Control Theory; Informal Social Control Theory	Parental phubbing was positively related to adolescent deviant peer affiliation which mediated adolescent mobile phone addiction.

Consistent with the findings of the first research question, the quality of parent-adolescent relationships significantly mediated the relationship between parental technoference and adolescent violent behaviours. One study found that adolescents who perceived lower levels of maternal acceptance were more likely to be cyberbully perpetrators [52]. Similarly, decreased perceptions of parental warmth was found to predispose adolescent cyberbullying perpetration [40]. Further, it was reported that adolescent-parent attachment style moderated the association between parental technoference and deviant peer affiliation [48]. Studies also identified potential psychological factors which influenced the relationship between parental technoference and adolescent violent behaviours. Adolescents who reported higher levels of anxiety were found to be more likely to cyberbully others [50], whilst emotional stability was also identified as a mediating component [52]. Further, it was found that adolescent moral disengagement and online disinhibition significantly exacerbated the relationship between parental technoference and cyberbullying perpetration [51].

## Discussion

Impact of parental technoference and phubbing has increased as the presence of technology in day-to-day life has increased. This review summarised the evidence exploring associations between parental technoference and adolescent mental health and/or violent behaviour. This review is the first to examine evidence on parental technoference and adolescent outcomes. The authors of this paper interpreted their results in the light of four key theories described within the included papers. Displacement hypothesis explains how parents may replace social and emotional interactions with their adolescent with their digital device instead [42, 45] whilst attachment theory is relevant to understand that when parents prioritise their device over adolescents’ emotional needs, it could lead to feelings of neglect and insecurity within adolescents [40]. Given that adolescents rely on their parents for emotional support, guidance and reassurance, parental technoference can lead to feeling of emotional neglect [53]. Adolescents may perceive their parents pre-occupation with their digital device as a lack of interest or disengagement, impacting the development of a secure attachment. Frustration aggression theory describes the displaced aggression adolescents may exhibit as a result of parental technoference [51]; and social learning theory underpins how adolescents may observe and imitate their parents’ technology habits [46, 49]. The review identified limited studies (N = 13) exploring the association between parental technoference and adolescent mental health and violent behaviour. Nonetheless, overall, findings from the identified studies consistently suggest that parental technoference can contribute to poorer levels of adolescent mental health and increased violent behaviours. These are salient findings given that technology is ever encroaching within family life. Adolescents recognise that occasional parental technoference is a normative part of living within a digital society [40]. Results suggest that lower levels of perceived parental technoference, defined as relatively minor and infrequent experiences, may have minimal impact on the mental health or violent behaviour of young people, however, persistent perceptions of parental technoference correlated with poorer mental health outcomes and increased violent behaviour. Therefore, the present review indicates that contextual factors, including the frequency and duration of use, are of high importance.

The literature identified in the present review illustrates the indirect influence of parental technoference on adolescent mental health and violent behaviour. A common interpretation within the studies herein is that electronic devices are not the direct cause of poor mental health or violent behaviour among adolescents, but rather an indirect consequence of the parent adolescent relationship, beyond digital devices [14]. Eligible studies reported that adolescent experiences of frequent parental technoference is associated with decreased perceptions of parental sensitivity and warmth and increased perception of parental rejection, which is related to negative emotions such as depression, anxiety, and addictive and violent behaviour. An explanatory model for the association between parental technoference and the parent-adolescent relationship is the displacement hypothesis [54], which in this context proposes that time spent on digital devices displaces time spent with other individuals. In reference to our first research question exploring the association between parental technoference and the mental health of adolescents, this theory would suggest that prolonged time spent on a digital device reduces opportunities to show sensitive parenting and sustain attuned parent-child interactions. When parents frequently allow digital devices to distract them from interacting with their adolescent, it is possible that the adolescent may perceive the parents as less responsive and supportive, which in turn can discourage feelings of cohesion; a crucial determinant of parent-adolescent attachment quality [55]. The adolescent-parent bond is one of the most pivotal bonds to be formed and the characteristics of the attachment play a critical role in adolescent outcomes, which can continue into adulthood. A substantial body of research has reported that diminished parent-adolescent cohesion and low satisfaction in family functioning is strongly associated with poorer adolescent mental health [56–58]. In the case of parental technoference, parental neglect for their adolescent’s needs for cohesion can exasperate adolescent perceptions of rejection, resulting in lower self-evaluation and increasing vulnerability to poorer mental health. Subsequently, the findings of the present review suggest that parental technoference is indirectly associated with poor mental health and decreased well-being through parent adolescent relationships. With the continuous growth of technology [59], future research examining these associations could inform practical guidelines for safeguarding the parent-adolescent relationship from the consequences of parental digital device use.

Our review identified only five studies measuring associations between parental technoference and adolescent violent behaviours, and these predominantly explored cyberbullying. The results consistently indicated that parental technoference significantly predicted adolescent cyberbullying perpetration. Technoference as an excluding behaviour is described as sending a direct message to adolescents that digital devices take precedence over spending time with them [60], thus leading to feelings of rejection or neglect. This impression can elicit feelings of frustration when regularly faced with parental technoference. From this perspective, adolescents may be more likely to engage in displaced aggression, such as bullying blameless victims online. This can be explained by the frustration aggression theory [61], which postulates that adolescents become so disconcerted at feeling rejected by their parents they retaliate in the form of tormenting others. Accordingly, the results of this review suggest that the quality of the family environment may increase new forms of aggression in the digital age such as cyberbullying. Studies herein also identified parental technoference as a potential risk factor to deviant peer affiliation. Given the significance of peer influence during adolescence, young people are likely to adhere to the pressures of deviant associates [62]. Previous research has identified that adolescent alliances with individuals who exhibit delinquent behaviours increase the development of deviant and antisocial undertakings [63, 64]. It has also been advocated that adolescent deviant peer affiliation is strongly influenced by negative factors within the immediate environment, in particular, the relationship between parent and adolescent [65]. Subsequently, taking into consideration that parental technoference has the potential to interrupt the attachment between parent and adolescent, which is a protective factor in deviant peer affiliation, it is possible that associating with deviant peers is an attempt by adolescents to gain emotional support they are lacking from their parents.

A direct connection between parental technoference and adolescent mental health and violent behaviour is also presented within the review. The results suggest that parents may be directly modelling unfavourable technological habits which are replicated by adolescents, for example, high frequency parental technoference predicted addictive digital device behaviours among adolescents. Moreover, studies found a positive correlation between parental technoference and adolescent technoference and that these behaviours can subsequently both independently and collaboratively influence adolescent depression, anxiety and cyberbullying [40, 42]. This direct effect could be explained by the social learning theory [66], which states that children model parental behaviours. That is, adolescents will acquire unhealthy digital device habits by observing and imitating the behaviours of their parents. Similarly, the relationship between parental technoference and adolescent cyberbullying could be related to parents modelling aggressive behaviours [26]. Previous research has found that parents are more hostile and respond harshly toward their children when interrupted in their device use [22]. Potentially, these parental attitudes may be replicated by adolescents and transferred into alternative environments leading to angry or hostile behaviour towards others. Given that parents are prominent role models to adolescents [67], the findings of this review are important to inform parents on the significant role they play in their adolescent’s behaviours.

The current review also acknowledges that not all adolescents homogenously experience the impact of parental technoference. Identified studies reported potential mechanisms that mediate the robustness of the association between parental technoference and adolescent outcomes.

Results indicated that poorer adolescent mental well-being was associated with greater sensitivity to perceived parental technoference and was further related to poorer mental health and violent behaviours. These findings may suggest that adolescents with higher levels of mental well-being are less inclined to interpret parental technoference negatively or as a form of parental rejection, and subsequently are less impacted by the experience.

Theoretically, our review suggests that parental technoference can negatively impact adolescent mental health and violent behaviours indirectly through diminishing the quality of parent-adolescent attachments. Practically, the findings indicate that parents should be encouraged to be aware of the environment they use electronic devices in and how this use can directly and indirectly impact adolescent health and behaviours. Given the benefits of digital devices within daily life, strategies must be found for using electronic devices in a way that minimise the harm they may cause on those around. Currently, recommendations on parental management of device use during family time are scarce [22]. It is argued that disconnecting from devices will become increasingly difficult as the use of technology continues to grow [59], thus, exploring the caveats of parental technoference to the parent-adolescent relationship will be crucial to establish recommendations to parents on the use of technology within the family context. Examining young people’s perceptions of how their own technology use may impact their interactions with others is another future direction for this research.

A future research programme could provide evidence-based practical and achievable guidelines for parents’ digital device usage. Technology can be considered as another part of the family environment, and developing practices to minimise the negative consequences it can elicit should be considered. As such the development of guidelines to raise awareness of parental interactions with technology and how this can impact family dynamics is important, and could include suggestions on limiting their device use whilst in the presence of their child and establishing media boundaries such as adopting the American Academy of Paediatrics Family Media Use Plan [68]. However, the acceptability of such guidelines should also be considered.

To date, the views of adolescents have been relatively unexplored when investigating parental technoference [40]. However, given that adolescents feel discontentment at persistent parental technoference, obtaining their perspective is important when investigating adolescent outcomes. Furthermore, understanding youth attitudes towards parental use would contribute to one of the main principles of the United Nations Sustainable Development Goals [69], which aims to promote the well-being of all individuals with a focus on preventable problems.

This review shows potential psychological factors which played an active role in how parental technoference impacted adolescent mental health and violent behaviours. Future research focusing on identifying mechanisms that could exasperate the effect of perceived parental technoference could identify adolescents most vulnerable to parental technoference. Identifying those most susceptible to the negative effects of parental technoference provides the opportunity to construct resilience-building strategies within adolescents which should lead to improved outcomes in later life. Furthermore, researchers have begun to examine the reasons for parental digital device use and the impact on children, identifying a link between parental depression and parenting stress and increased parental technoference during parent-child interactions [16]. However, the moderating role of parental characteristics remains unclear in the association between parental technoference and adolescent outcomes, and future studies could investigate confounding components, such as parental mental health, well-being, income, education level and work-life balance.Identified studies exploring the association between parental technoference and violent behaviours primarily focused on online violence in the form of cyberbullying perpetration. Given that previous studies have reported harsher parenting styles when children disrupt electronic device use, the aggressive attitudes observed by adolescents may be replicated and transferred into other areas in their lives. Correspondingly, emerging research demonstrates a positive association between parental technoference and offline violent behaviours in the form of physical aggression in children aged 5–10 years [26]. However, our review found no studies exploring relationships between parental technoference and adolescent aggression, highlighting a critical gap in the literature.

### Limitations

While our review used systematic searching and data extraction methods, the analysis was limited due to the scarcity of evidence concerning the subject under investigation. Additionally, contrasting research in this topic for young children has been conducted in the USA whilst research on adolescents has predominantly taken place in China, which limits the generalisability of the present findings to other countries and demonstrates the need for studies across broader geographies to subsequently inform guidelines for families surrounding the use of technology within the household.

The limitations of the included studies should also be considered when interpreting the conclusions of this review. First, a primary limitation within the included studies is that adolescents self-reported on digital device use and their mental health and violent behaviours, which may be biased. Adopting objective measures of technology use would decrease potential bias and allow future research to be more robust. Second, although associations between variables have been provided, as well as proposed mediating factors, the cross-sectional design of the included studies limits the opportunity to explore causal relationships. Future longitudinal research would allow the evaluation of potential risk factors between parental technoference and adolescent outcomes by analysing the characteristics of participants over time. Third, the infrequent reporting of the extent of exposure to parental technoference within the included studies (n = 2 articles) also restricted understanding of the potential association between technoference on adolescent outcomes. Nonetheless, despite these limitations, to our knowledge, the current review is the first to collate literature surrounding the association between parental technoference and adolescent mental health and violent behaviour and address gaps within the literature.

## Conclusion

Our review aimed to identify existing literature exploring the association between parental technoference and adolescent mental health and violent behaviour. Findings suggest that parental technoference may contribute to poorer mental health and increased violent behaviours in adolescents. However, major gaps in evidence exist. The findings indicate that parental technoference may be associated with parental unresponsiveness, thus suggesting that parents should be encouraged to be aware of the environment in which they use electronic devices and how this can directly and indirectly influence adolescent health and behaviour. Further research into the caveats of parental technoference is needed to inform guidelines for family management of devices to ensure the health and well-being of adolescents. The review also highlights potential psychological factors which play an active role in how parental technoference can potentially impact adolescent mental health and violent behaviours. Future investigations into the underlying mechanisms and moderating factors would contribute to identifying those who are more vulnerable to parental technoference.

### Implications and contribution

Further research is required to inform the development of evidence-based parental guidelines on raising children in a digital household. The current scoping review is the first to identify studies which specifically explore the association between parental technoference and adolescent mental health and violent behaviour.

### Electronic supplementary material

Below is the link to the electronic supplementary material.

Supplementary Material 1

## Data Availability

The datasets used and analysed during the current study are available from the corresponding author on reasonable request.
